# Objectively Measured Neighborhood Walkability and Change in Physical Activity in Older Japanese Adults: A Five-Year Cohort Study

**DOI:** 10.3390/ijerph15091814

**Published:** 2018-08-22

**Authors:** Hiroyuki Kikuchi, Tomoki Nakaya, Tomoya Hanibuchi, Noritoshi Fukushima, Shiho Amagasa, Koichiro Oka, James F. Sallis, Shigeru Inoue

**Affiliations:** 1 Department of Preventive Medicine and Public Health, Tokyo Medical University, 6-1-1 Shinjuku, Shinjuku-ku, Tokyo 160-8402, Japan; fukufuku@tokyo-med.ac.jp (N.F.); amagasa@tokyo-med.ac.jp (S.A.); inoue@tokyo-med.ac.jp (S.I.); 2. Graduate School of Environmental Studies, Tohoku University, 468-1 Aoba, Aramaki, Aoba-ku, Sendai 980-0845, Japan; tomoki.nakaya.c8@tohoku.ac.jp; 3 School of International Liberal Studies, Chukyo University, 101 Tokodachi, Kaizu-Cho, Toyota 470-0393, Japan; info@hanibuchi.com; 4 Faculty of Sport Sciences, Waseda University, 2-579-15 Mikajima, Tokorozawa 359-1192, Japan; koka@waseda.jp; 5 Family Medicine and Public Health, University of California San Diego, 9500 Gilman Drive, La Jolla, CA 92093-0631, USA; jsallis@ucsd.edu; 6 Mary MacKillop Institute for Health Research, Australian Catholic University, Level 5, 215 Spring Street, Melbourne 3000, Australia

**Keywords:** exercise, built environment, neighborhood environment, prospective cohort study, geographic information system (GIS)

## Abstract

Objectives: This study investigated the longitudinal association between changes in older adults’ physical activity and neighborhood walkability measured by geographic information systems (GISs, (ArcGIS, ESRI Inc., Redlands, CA, USA)). Methods: A mail survey was conducted for Japanese older adults who were randomly selected from three different settlement types. Data on walking, total moderate to vigorous physical activity (MVPA), and sociodemographic characteristics were collected at baseline (in 2010) and follow-up (in 2015). Multiple linear regression analyses were employed to assess the association between MVPA change and neighborhood walkability, adjusted for potential confounders. Effect sizes for independent variables on MVPA change were estimated. Results: Data from 731 community-dwelling older adults (43.7% women) were analyzed. During the follow-up, older adults’ MVPA was reduced by 94.4 min/week (−14.2%) on average (675.5 and 579.9 min/week in 2010 and 2015, respectively). Overall, older adults living in highly walkable areas showed a smaller reduction than those in low walkable areas (beta: 99.7 min/week, 95% confidence interval: 28.5–171.0). Similar associations were observed among those in the urban and suburban area, but not in the rural area. Walkability had larger effect sizes for explaining MVPA change than demographic characteristics. In addition, the findings for walking were similar to MVPA. Conclusion: Neighborhood walkability mitigated the 5-year reduction of walking and total MVPA among older adults, especially in urban areas.

## 1. Introduction

Increasing moderate to vigorous physical activity (MVPA) is considered an important strategy to reduce health risks globally [1,2]. Creating built environments that support physical activity is recommended by national [3] and international authorities [4]. There is strong evidence that neighborhood walkability, which is often derived from street connectivity, residential density, and land use mix [5,6], is related to individual MVPA not only among younger adults [7,8,9,10], but also older adults [11,12]. City planning to support walkability has been recommended to sustain or improve older adults’ MVPA for successful aging [13,14,15,16].

A recent systematic review of the relation between physical activity and built environment among older adults indicated that most (94%) of the studies had a cross-sectional design [12]. Cross-sectional study designs can suffer from reverse causation, with residential self-selection being a prominent example. That is, older adults who enjoy physical activity may be more likely to choose to live near a park, rather than parks stimulating more physical activity [12]. Thus, longitudinal study designs are important for providing stronger evidence regarding environmental causation.

In a recent systematic review [12], four studies examined the longitudinal association between built environment and physical activity among older adults [17,18,19,20]. However, these studies were mostly limited to urban areas in North American countries. Since urban–rural differences in older adults’ physical activity levels have been documented [21,22], it is possible that built environments in rural areas impact differently on physical activity, compared to urban areas. Sallis et al. indicated the importance of studies ensuring wide variability in environments and populations to avoid underestimation of true associations between physical activity and built environments [23]. Therefore, longitudinal studies are needed to improve evidence on the association between neighborhood walkability and physical activity levels among older adults living in wide range of urbanity.

The present study aimed to investigate the association between five-year changes in physical activity and neighborhood walkability measured using geographic information systems (GISs), among community-dwelling older adults in urban, suburban, and rural areas.

## 2. Materials and Methods

### 2.1. Study Design and Data Collection

This population-based cohort study was conducted using a postal survey in 2010 (baseline) and 2015 (follow-up). For the baseline survey in 2010, detailed sampling procedures are described elsewhere [24]. Briefly, the study was carried out in three Japanese municipalities; Bunkyo Ward (an urban city in the Tokyo metropolitan area); Fuchu City (a suburban city of Tokyo within commuting distance to the central business district); and Oyama Town (a typical small rural area located about 80 km west of Tokyo). Older adults aged between 65 and 74 years were randomly selected from the registry of residential addresses of each municipality, stratified by gender and by age (65–69 years and 70–74 years). In total, 2700 community-living older adults were identified. Finally, 2046 participants returned the survey (response rate 75.7%).

For the follow-up survey in 2015, detailed sampling procedures are also described elsewhere [25]. Of 2046 people who returned the questionnaire at baseline, 1314 agreed to complete the survey at follow-up. In 2015, we sent an advance notification of the intention to send a questionnaire 1 month before the second survey, and 104 participants were excluded due to inability to contact or death. Therefore, we sent a questionnaire to 1210 participants at follow-up, and 956 participants returned the questionnaire. Among these respondents, 161 who had any difficulty performing daily activities assessed by the Japanese 8-item Short-Form Health Survey (SF-8) [26] were excluded from the present analyses. In addition, we excluded 64 due to missing data of physical activity (*n* = 40) and missing data on other variables in the analyses (*n* = 24). Therefore, there were 731 (410 men, 321 women) final participants.

Ethical approval for the study was obtained from the Tokyo Medical University Ethics Committee prior to the survey in 2010 (No. 1273) and in 2015 (No. 2898). All participants signed the consent form before answering the questionnaire.

### 2.2. Dependent Variable: 5-Year Changes in Walking and Total MVPA Time

Data on walking and total MVPA were collected at both time points using the Japanese version of the International Physical Activity Questionnaire (short version) [27,28]. Participants were asked to report the frequency and duration of three categories of physical activity: vigorous intensity, moderate intensity (excluding walking), and walking. Total time (min/week) spent in walking and MVPA, including walking, was calculated by adding these three activities. Change of walking and total MVPA time over the 5-year period was calculated by subtracting MVPA time at follow-up from time at baseline survey.

### 2.3. Independent Variables: Neighborhood Walkability

In this study, neighborhood was defined as a polygon-based street-network buffer around each respondent’s home address, which was geocoded at the level of small city block (Banchi or Go). Neighborhood walkability score was calculated by combining three built environment measures; i.e., residential density, street connectivity and land use mix within each neighborhood polygon. These three features of neighborhood are commonly studied for their associations with physical activities [6,10,29]. Given that the relevant size of a neighborhood could vary according to the age group, we considered a street network distance of 500 m as representing the easily accessible space for an ambulatory older adult. A street network distance of 1000 m was also considered because de Sa and Ardern indicated that the association between neighborhood walkability and transport-related physical activity differed by 500-m and 1000-m buffer zones [30]. We used ArcGIS 9.3 software with Network Analyst Extension (ESRI Inc. (Redlands, CA, USA)) for all spatial calculations.

### 2.4. Independent Variables: Neighborhood Walkability

#### 2.4.1. Residential Density

Gross population density was calculated as estimated residential population size divided by the areal size of the neighborhood polygon based on building square meters. The 500-m gridded census-population as of 2005 was proportionally divided into each neighborhood based on the proportion of area sizes of overlapped spaces between the population grid and residential areas within the neighborhood. With the Digital Map 2500 (Spatial Data Framework), published by the Geospatial Information Authority of Japan (GSI), we used only built-up area as the residential area for the population estimation by excluding water bodies (rivers, lakes, and ponds) and parks. In the case of Oyama town, detailed housing footprints in the dataset of the Fundamental Geospatial Information provided by GSI were available, so we used them for the proportional distribution of gridded population data into each neighborhood. It should be noted that in all of the three cities, the GIS-measured geometrical area size of the buffer was used for calculating the population density value.

#### 2.4.2. Street Connectivity

Intersection density, which was derived by the number of street intersections (at least three-way) in each buffer area divided by buffer area (square kilometers), was used as an index of street connectivity. The information was obtained from the Digital Map 25000 (Spatial Data Framework).

#### 2.4.3. Land-Use Mix

The number of local destinations in each buffer was used to measure the land use mix. Considering previous studies as well as the Japanese context, we chose five common destinations: convenience stores, supermarkets, post offices, clinics, and parks. The data on convenience stores and supermarkets were obtained from DARMS 2007 (JPS Inc. which became Zenrin Geo Intelligence Co., Ltd. in 2016, Tokyo, Japan), a large GIS dataset for marketing. The data on post offices were given by the National Land Numerical Information, while the locations of entrance points to parks were manually identified by browsing various internet-based map services such as Mapion (Mapion Co., Ltd., Tokyo Japan). The data on clinics and hospitals were obtained from PAREA Medical 2008, and the database of medical facilities was provided by Kokusai Kogyo Co., Ltd.

#### 2.4.4. Scoring of Neighborhood Walkability

Based on previous studies [31,32], we created a single index for neighborhood walkability by calculating the sum of the standard scores of the three built environment measures. For population density and street connectivity, the standard scores were derived from calculating z-scores using population density and street intersections density. For the standard score of land-use mix, we counted the total number of five destinations (consisting of supermarkets, convenience stores post offices, clinics and parks), and then the z-score was calculated. Finally, neighborhood walkability scores were derived by summing up these three z-scores.

### 2.5. Covariates

Information about age, gender, and residential area were obtained from the resident registry of each municipality. The following variables acquired through self-reported questionnaires were relevant confounders for statistical control: living arrangement (living alone or living with others), working status (currently working or not), body mass index (BMI; lea (less than 18.5 kg/m^2^), normal (18.5–24.9 kg/m^2^) or overweight (25.0 kg/m^2^)), self-rated health (good or poor) and driving status (driver or non-driver). Smoking (current smoker or not) and alcohol drinking (at least once per month or not) status was examined using items from the National Health and Nutrition Survey in Japan.

### 2.6. Statistical Analyses

The distribution of walkability score by area of residence was visualized by using box-whisker plots [33]. Multivariate regression analyses were used for the association between neighborhood walkability and 5-year change of MVPA, adjusting for age, gender, living arrangement, educational attainment, employment status, current smoking status, and alcohol intake. All analyses were conducted separately by a 500-m or 1000-m network buffer. Then stratified analyses by area of residence were completed. In each regression analysis, effect sizes of independent variables on MVPA change were estimated by calculating eta-squared [34]. We used STATA software (version 13; Stata Corp, College Station, TX, USA); the level of significance was set at 0.05.

## 3. Results

**Figure 1:**
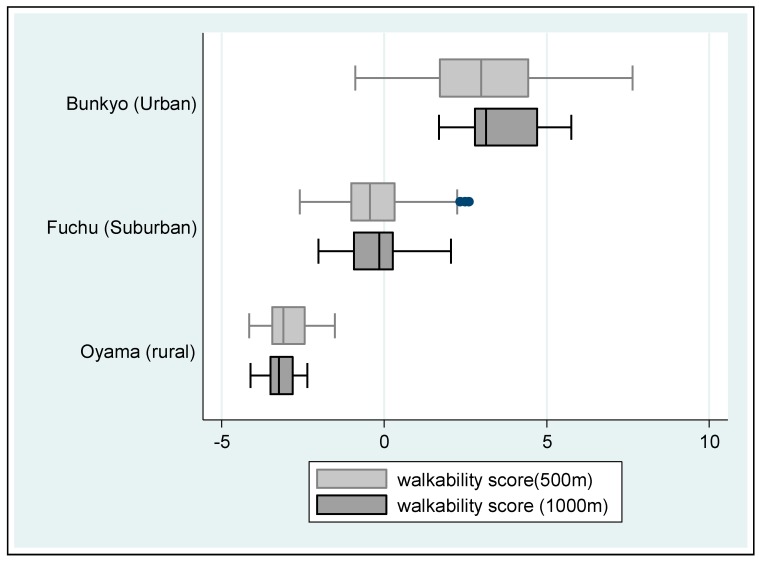
Distribution of individual walkability score by city of residence Note: Box-whisker plot indicates median line, quartile box, whiskers for the most extreme cases inside 1.5× interquartile range (IQR), and outliers.

Table 1 shows baseline characteristics of study participants. Data from 731 community-dwelling older adults (43.7% women) were analyzed. During the 5-year follow-up, older adults’ walking and MVPA time was reduced by 57.0 (−12.6%) and 94.4 min/week (−14.2%) on average, respectively. Figure 1 illustrates the distribution of walkability score by area of residence, revealing large differences.

Table 2 and Table 3 show the results of multivariate regression analyses for change of MVPA and walking, respectively. As shown in Table 2, men, urban residents, employed participants and those with overweight had less decline in 5-year MVPA than other subgroups. In addition, older adults living in highly walkable areas showed smaller reductions in MVPA than those in low walkable areas for both network buffer sizes (500-m network buffer: beta = 55.7 min/week, 95% confidence interval (CI): 4.7–106.8, 1000-m network buffer: beta = 97.0, 95% CI: 25.8–168.2). Effect sizes for urban residence and walkability on MVPA change were relatively larger than other independent variables. As shown in Table 3, older adults living in highly walkable areas showed smaller reductions in walking time than those in low walkable areas for the 1000-m network buffer (beta = 47.5, 95% CI: 1.6–93.4).

Table 4 showed the result of stratified analyses by area of residence. In total, neighborhood walkability mitigated the 5-year decline of MVPA and walking for the 1000-m network buffer. The stratified analyses showed this association were observed more clearly among those in the urban area (MVPA: 500-m network buffer: beta = 78.4, 95% CI: 13.0–143.9, 1000-m network buffer: beta = 113.3, 95% CI: 13.6–213.0, walking: 500-m network buffer: beta = 42.7, 95% CI: 6.8–78.6, 1000-m network buffer: beta = 62.2, 95% CI: 7.5–116.8). In addition, marginal associations were also found among those in the suburban area (1000-m network buffer: beta = 98.2, 95% CI: −10.1 to 206.5). However, no associations were found among those in rural area. Compared to rural area, relatively larger effect sizes for walkability on MVPA change were observed in urban or suburban areas. In addition, the findings for walking were similar to MVPA.

## 4. Discussion

This study examined the longitudinal association between GIS-measured neighborhood walkability and 5-year changes in older adults’ physical activity. Overall, older adults’ MVPA declined by 14.2% over 5 years, but those living in higher walkable areas showed significantly smaller reductions than those in lower walkable areas. In stratified analysis by area of residence, higher neighborhood walkability mitigated the reduction of MVPA among older adults living in urban or suburban areas. However, significant associations were not observed among those in the rural area. Present findings support the importance of walkable city planning for sustaining MVPA levels among older adults, especially in urban and suburban areas.

A recent systematic review showed that there were 94 cross-sectional and five longitudinal studies (including one doctoral thesis) which investigated the association between built environment and physical activity among older adults [12]. In four longitudinal studies published in scientific journals, one used subjective measures [20] and three used objective measures for assessing neighborhood built environment [17,18,19]. Two studies used GISs [17,19], and the other one used an unidentified commercial software [18]. In summarizing the two GIS-based longitudinal studies, one study of 513 Americans in Portland city showed that older men living in areas with more recreational facilities or parks had relatively lower reduction in walking time over five years [19]. The other study of 521 Canadians in Montreal or Laval City showed that proximity to services or amenities (i.e., mixed land use) was associated with higher frequency of walking at all times over three years [17]. Both past studies conducted in urban cities and showed walkability-related variables mitigated the decline of physical activity in older adults. Thus, present findings were generally consistent with the two similar prior studies. However, the present study suggested new findings for rural areas; that is, there may be little association between built environment and physical activity change.

It is notable that walkability or residential area had the highest effect sizes for explaining MVPA change; more than well-documented influential individual factors such as sex or age [35,36]. That may imply that environmental attributes have considerable effect on MVPA decline among older adults. Thus, this study empirically suggests the importance of creating built environment to sustain older adults’ MVPA, which is recommended by national [3] and international authorities [4].

The association between high walkability and less MVPA reduction was significant among older adults living in Bunkyo (urban) and Fuchu (Suburban) city, whereas the association was not seen in Oyama (rural) town. The reason for these urban-rural differences were not directly addressed by the study, but the following explanations are offered, based on conceptual models and prior findings. First, the narrower distribution of walkability scores in Oyama (rural) town reduced statistical power to detect an association. Second, within the lower range of walkability, some improvement in neighborhood walkability (i.e., “very low” walkable to “low” walkable) may not be sufficient to support older adults’ MVPA. By contrast, improving walkability in higher ranges of walkability (i.e., “high” walkable to “very high” walkable) would be expected to be positively related to more physical activity. Providing some support for this idea, a longitudinal Canadian study found more favorable obesity and type 2 diabetes trends only among adults living in the 20% most walkable areas compared to all other walkability levels [37]. Third, the walkability concept may not apply well to rural areas, such as farms, that are, by definition, of low density, with low connectivity and few destinations within walking distance. However, walkability is expected to apply similarly across a range of urbanity from big cities to small towns. In an international study of adults that did not include rural areas, walkability was positively and linearly related to objectively assessed MVPA [38]. Further research is needed to identify environmental attributes related to physical activity among rural-dwelling older adults.

Compared with rural area where almost all neighborhoods are uniformly low walkable, it may be more feasible to improve walkability in urban areas where higher and lower walkable areas were proximal to each other. A further benefit of intervention in urban and suburban areas is that built environment changes covering relatively small areas would affect more people due to higher residential density, making such changes more cost-effective. Currently, the World Health Organization (WHO) recommends environmental interventions to sustain older adults’ MVPA [13,14]. Such interventions may be more effective in urban areas.

The results in both combined analyses (Table 2 and Table 3) and area-specific analyses (Table 4) indicated that neighborhood environment effects were stronger using 1-km buffers as compared to 500-m buffers. This finding suggests that residents can be influenced by attributes using the larger definition of neighborhood and that walking 1 km or more in the neighborhood is likely to be common. Sugiyama et al. showed that local destinations within a 10-min walking distance in Australia, and 20-min walking distance in Japan were associated with walking for transport [37]. Taken together, the implication is that the potential impact of neighborhood sizes on MVPA could vary by country, culture, or degree of urbanity.

The present study had several strengths. Community-dwelling older adults were randomly selected from three different localities (urban, suburban and rural) with very different land use patterns. The longitudinal design enabled more confident interpretations of the causality between neighborhood walkability and MVPA change. However, some limitations of our study should also be considered. First, physical activity was assessed by self-report measures. The use of objective assessment for measuring MVPA would be more desirable in future studies [39]. Second, the self-report physical activity measure did not provide domain-specific data, and there is strong evidence that various built environment attributes are related differently to leisure, transport, and occupational domains of physical activity [40]. Domain-specific analyses may have provided different results, because walkability is most consistently related to active transportation [5,12]. Third, the follow-up rate was limited because many participants who responded to the baseline survey declined to complete follow-up surveys, and others had missing data. Fourth, although we adjusted for several possible confounding variables, there may have been unmeasured confounding variables. Lastly, there are other environmental aspects which have been reported as cross-sectional correlates of older adults’ MVPA that were not measured in the present study. These variables include access to recreational facilities [39,40] or traffic/crime safety [41,42]. Future studies need to include these variables in longitudinal study studies.

## 5. Conclusions

In conclusion, this prospective study found higher neighborhood walkability was significantly associated with smaller declines of MVPA among older adults, especially among residents of urban and suburban areas. Walkability and area of residence had larger effect sizes for explaining MVPA change than demographic characteristics including age and sex. Present findings with older Japanese adults were generally consistent with prior studies from other countries [17,18,19,20]. This growing literature provides evidence to support international recommendations promoting built environment changes to create more walkable neighborhoods to facilitate more active and healthy aging [38,43].

## Figures and Tables

**Table 1:** Baseline characteristic of participants.

Variable	Total		Urban (Bunkyo)		Suburban (Fuchu)		Rural (Oyama)	
	*n* or Mean	% or SD	*n* or Mean	% or SD	*n* or Mean	% or SD	*n* or Mean	% or SD
Gender								
Men	410	(55.8%)	127	(54.0%)	148	(59.4%)	135	(54.7)
Women	321	(43.7%)	108	(46.0%)	101	(40.6%)	112	(45.3)
Age (years)	69.3	(2.9)	69.0	(2.9)	69.1	(0.3)	69.8	(3.0)
Living arrangements								
Living with others	664	(90.3%)	206	(87.7%)	227	(91.2%)	231	(93.5)
Living alone	67	(9.1%)	29	(12.3%)	22	(8.8%)	16	(6.5)
Educational attainment								
College degree or more	304	(41.4%)	138	(58.7%)	114	(45.8%)	52	(21.1)
Up to high school	427	(58.1%)	97	(41.3%)	135	(54.2%)	195	(78.9)
Employment status								
Yes	332	(45.2%)	125	(53.2%)	102	(41.0%)	105	(42.5)
No	399	(54.3%)	110	(46.8%)	147	(59.0%)	142	(57.5)
Current driver								
Yes	371	(50.5%)	72	(30.6%)	110	(44.2%)	189	(76.5)
No	360	(49.0%)	163	(69.4%)	139	(55.8%)	58	(23.5)
Current smoker								
Yes	114	(15.5%)	30	(12.8%)	42	(16.9%)	42	(17.0)
No	617	(83.9%)	205	(87.2%)	207	(83.1%)	205	(83.0)
Alcohol intake								
Yes	352	(47.9%)	104	(44.3%)	111	(44.6%)	137	(55.5)
No	379	(51.6%)	131	(55.7%)	138	(55.4%)	110	(44.5)
Self-rated health								
Good	668	(91.4%)	218	(92.8%)	221	(88.8%)	229	(92.7%)
Poor	63	(8.6%)	17	(7.2%)	28	(11.2%)	18	(7.3%)
Body mass index								
Leanness (<18.5)	39	(5.3%)	14	(6.0%)	12	(4.8%)	13	(5.3%)
Normal (18.5–24.9)	555	(75.9%)	174	(74.0%)	196	(78.7%)	185	(74.9%)
Overweight (≥25.0)	137	(18.7%)	47	(20.0%)	41	(16.5%)	49	(19.8%)
Walking (min/week)								
at baseline (2010)	451.9	(475.4)	432.8	(412.7)	423.0	(442.9)	499.3	(554.5)
at follow-up (2015)	395.0	(436.3)	383.8	(371.6)	378.5	(427.2)	422.2	(498.4)
5-year change	–57.0	(550.0)	–49.0	(470.9)	–44.5	(522.8)	–77.1	(640.4)
% change	–12.6%		–11.3%		–10.5%		–15.4%	
MVPA (min/week)								
at baseline (2010)	675.5	(799.6)	659.5	(784.6)	624.7	(711.4)	742.0	(891.1)
at follow-up (2015)	579.9	(668.8)	537.4	(539.1)	592.2	(729.6)	607.9	(715.3)
5-year change	–94.4	(861.9)	–122.1	(883.7)	–32.5	(798.1)	–134.1	(907.9)
% change	–14.2%		–18.5%		–5.2%		–18.1%	

Note: MVPA: moderate to vigorous physical activity.

**Table 2:** Association between change of total MVPA in the 5-year period and neighborhood walkability/socio-economic status: Multivariate regression analyses.

Variable	500-m Network-Buffer				1000-m Network-Buffer			
	Beta (min/week)	(95% CI)	*p*	Effect Size (Eta-Squared)	Beta (min/week)	(95% CI)	*p*	Effect Size (Eta-Squared)
Gender								
Men	(ref)				(ref)			
Women	**−158.7**	**(−316.2, −1.1)**	**0.048**	**0.005**	−149.7	(−306.8, 7.4)	0.062	0.005
Age (years)	**25.8**	**(3.5, 48.1)**	**0.024**	**0.007**	**26.5**	**(4.2, 48.7)**	**0.020**	0.008
City of residence								
Urban (Bunkyo)	−223.7	(−457.1, 9.7)	0.060	0.004	**−385.5**	**(−686.7, −84.3)**	**0.012**	0.007
Suburban (Fuchu)	(ref)				(ref)			
Rural (Oyama)	64.7	(−146.1, 275.4)	0.547	0.001	201.5	(−62.3, 465.3)	0.134	0.003
Living arrangements								
Living with others	(ref)				(ref)			
Living alone	90.0	(−129.6, 309.5)	0.421	0.001	88.0	(−131.0, 307.1)	0.430	0.001
Educational attainment								
College degree or more	(ref)				(ref)			
Up to high school	−1.0	(−136.1, 134.1)	0.988	<0.001	7.0	(−128.1, 142.1)	0.919	<0.001
Employment status								
Part-time or full-time work	(ref)				(ref)			
Not working	**136.8**	**(4.4, 269.2)**	**0.043**	**0.006**	**140.7**	**(8.6, 272.9)**	**0.037**	**0.006**
Driving status								
Non-driver	(ref)				(ref)			
Driver	26.2	(−123.6, 176.1)	0.731	<0.001	38.0	(−111.9, 187.9)	0.619	<0.001
Current smoker								
No	(ref)				(ref)			
Yes	−82.0	(−262.8, 98.7)	0.373	0.001	−85.3	(−265.8, 95.2)	0.354	0.001
Alcohol intake								
No	(ref)				(ref)			
Yes	−139.1	(−279.9, 1.8)	0.053	0.005	−129.0	(−269.7, 11.6)	0.072	0.005
Self-rated health								
Good	(ref)							
Poor	88.3	(−136.2, 312.9)	0.440	0.001	86.8	(−137.3, 311.0)	0.447	0.001
Body mass index								
Leanness (<18.5)	−157.6	(−440.4, 125.3)	0.274	0.002	−161.6	(−443.6, 120.4)	0.261	0.002
Normal (18.5–24.9)	(ref)							
Overweight (≥25.0)	**−170.6**	**(−331.5, −9.7)**	**0.038**	**0.006**	**−165.2**	**(−325.9, −4.6)**	**0.044**	**0.006**
Neighborhood walkability	**55.7**	**(4.7, 106.8)**	**0.032**	**0.006**	**97.0**	**(25.8, 168.2)**	**0.008**	**0.010**

Note: CI: confidence interval, MVPA: moderate to vigorous physical activity.

**Table 3:** Association between change of walking in the 5-year period and neighborhood walkability/socio-economic status: Multivariate regression analyses.

Variable	500 m Network-Buffer				1000 m Network-Buffer			
	Beta (min/week)	(95% CI)	*p*	Effect Size (Eta-Squared)	Beta (min/week)	(95% CI)	*p*	Effect Size (Eta-Squared)
Gender								
Men	(ref)				(ref)			
Women	**−105.5**	**(−206.8, −4.1)**	**0.041**	**0.006**	−100.7	(−201.9, 0.4)	0.051	0.005
Age (years)	12.4	(−2.0, 26.7)	0.091	0.004	12.7	(−1.6, 27.1)	0.082	0.004
City of residence								
Urban (Bunkyo)	−76.5	(−226.6, 73.7)	0.318	0.002	−148.4	(−342.4, 45.6)	0.134	0.004
Suburban (Fuchu)	(ref)				(ref)			
Rural (Oyama)	52.6	(−83.0, 188.2)	0.447	0.001	113.9	(−56.0, 283.8)	0.188	0.002
Living arrangements								
Living with others	(ref)				(ref)			
Living alone	31.2	(−110.1, 172.4)	0.665	0.000	30.9	(−110.1, 172.0)	0.667	0.000
Educational attainment								
College degree or more	(ref)				(ref)			
Up to high school	1.6	(−85.3, 88.5)	0.971	0.000	5.4	(−81.6, 92.4)	0.904	0.000
Employment status								
Part-time or full-time work	(ref)				(ref)			
Not working	52.9	(−32.3, 138.0)	0.223	0.002	54.9	(−30.2, 140.0)	0.206	0.002
Driving status								
Non-driver	(ref)				(ref)			
Driver	16.5	(−79.9, 112.9)	0.737	0.000	22.1	(−74.4, 118.7)	0.653	0.000
Current smoker								
No	(ref)				(ref)			
Yes	−17.9	(−134.2, 98.4)	0.762	0.000	−19.4	(−135.6, 96.8)	0.743	0.000
Alcohol intake								
No	(ref)				(ref)			
Yes	−75.9	(−166.5, 14.8)	0.101	0.004	−70.8	(−161.4, 19.8)	0.125	0.003
Self-rated health								
Good	(ref)							
Poor	54.7	(−89.8, 199.2)	0.458	0.001	53.8	(−90.6, 198.1)	0.465	0.001
Body mass index								
Leanness (<18.5)	−8.9	(−190.9, 173.1)	0.923	0.000	−11.6	(−193.2, 170.0)	0.900	0.000
Normal (18.5–24.9)	(ref)							
Overweight (≥25.0)	−75.3	(−178.8, 28.3)	0.154	0.003	−72.7	(−176.2, 30.8)	0.168	0.003
Neighborhood walkability	29.5	(−3.4, 62.3)	0.079	0.004	**47.5**	**(1.6, 93.4)**	**0.042**	**0.006**

Note: CI: confidence interval, MVPA: moderate to vigorous physical activity.

**Table 4:** Association between neighborhood walkability and change in total MVPA and walking during the 5-year period by area of residence: Multivariate regression analyses.

Variables	5-Year Changes (min/week)	Neighborhood Walkability							
		500 m Network-Buffer				1000 m Network-Buffer			
		Beta * (min/week)	(95% CI)	*p*	Effect Size (Eta-Squared)	Beta * (min/week)	(95% CI)	*p*	Effect Size (Eta-Squared)
**MVPA**									
Total	−94.4	**55.7**	**(4.7, 106.8)**	**0.032**	**0.006**	**97.0**	**(25.8, 168.2)**	**0.008**	**0.010**
Urban (Bunkyo)	−122.1	**78.4**	**(13.0, 143.9)**	**0.019**	**0.025**	**113.3**	**(13.6, 213.0)**	**0.026**	**0.022**
Suburban (Fuchu)	−32.5	36.2	(−145.6, 218.0)	0.695	<0.001	98.2	(−10.1, 206.5)	0.075	0.013
Rural (Oyama)	−134.1	10.2	(−83.0, 103.4)	0.829	<0.001	−71.3	(−348.8, 206.2)	0.613	0.001
**Walking**									
Total	−57.0	29.5	(−3.4, 62.3)	0.079	0.004	47.5	(1.6, 93.4)	0.042	0.006
Urban (Bunkyo)	−49.0	**42.7**	**(6.8, 78.6)**	**0.020**	**0.024**	**62.2**	**(7.5, 116.8)**	**0.026**	**0.022**
Suburban (Fuchu)	−44.5	13.5	(−47.2, 74.2)	0.661	<0.001	48.6	(−22.1, 119.3)	0.177	0.008
Rural (Oyama)	−77.1	−21.0	(−150.7, 108.8)	0.750	<0.001	−83.7	(−281.6, 114.2)	0.406	0.003

Note: CI: confidence interval, MVPA: moderate to vigorous physical activity. * Each beta indicates how MVPA decline in 5-years was changed by one increment of walkability index in each area. Adjusted covariates are age, gender, living arrangement, educational attainment, employment status, driving status, smoking status, alcohol intake, self-rated health and body mass index.
